# Energy Expenditure and Nutrition in Neurogenic Obesity following Spinal Cord Injury

**Published:** 2020

**Authors:** Gary J. Farkas, David R. Gater

**Affiliations:** 1 Department of Physical Therapy and Rehabilitation Science; University of California, San Francisco School of Medicine, San Francisco, CA 94143, USA; 2 Department of Anatomy; University of California, San Francisco School of Medicine, San Francisco, CA 94143, USA; 3 Department of Physical Medicine and Rehabilitation, Miami, FL 33136, USA

Worldwide, obesity is a public health concern and a metabolic ailment characterized by excessive adipose tissue accumulation resulting from an imbalance of energy expenditure and energy intake [1]. This disorder is a known risk factor for cardiovascular disease, type 2 diabetes mellitus, dyslipidemia, hypertension, and metabolic syndrome, and in recent years, it has been described as a systemic inflammatory disease with chronic consequences [2].

Over the past several years, numerous articles have published data and discussed concerns over the increased rate of obesity and/or metabolic dysfunction in individuals living with a spinal cord injury (SCI) [2–5]. As a consequence of the injury, the disruption of afferent and efferent spinal cord tracts lead to changes in whole-body homeostasis that increases the risk of morbidity and mortality [2]. Both complete and incomplete injuries experience a rapid loss of contractile proteins that manifests through an obligatory sarcopenia, a few weeks after the injury with a continued reduction up to a year following the SCI. This is exacerbated by greater time since injury, injury completeness, and level of injury [2]. Concurrent with atrophy of skeletal muscle, there is a substantial loss of bone mineral content due to mechanical unloading, anabolic insufficiency, sympathetic nervous system dysfunction, and loss of neurotrophic influences [5]. This increases rates for osteoporosis and fracture below the level. These changes in body composition, specifically an obligatory sarcopenia, contribute to a significant decrease in total daily energy expenditure (TDEE) and an accumulation of adipose tissue, referred to as neurogenic obesity [2].

TDEE is equal to the total of the thermic effect of food (TEF), the thermic effect of physical activity (TEPA), and basal metabolic rate (BMR) [5,6]. The TEF is the least variable constituent of TDEE since absorption and digestion of food remains constant, and is relatively uninfluenced by body weight and body composition. TEPA is the most variable part of TDEE, and is dependent on lean body mass and adjustable parameters of physical activity (i.e., intensity, frequency, duration, and mode). BMR contributes the greatest to TDEE and is the minimum energy needed to support cellular metabolism and processes [6]. Fat free mass (FFM), composed of bone, muscle, and organs, contributes the most to BMR, and of FFM, skeletal muscle mass accounts for 85% of the variance. Following a SCI, with the loss of metabolically active tissue, BMR is significantly decreased, which results in a proportionate loss in TDEE by over 50% in persons with tetraplegia. Accurate determination of BMR is essential given its use in estimating TDEE and in prescribing appropriate energy intake recommendations to individuals with SCI [2,5].

Many equations and activity correction factors used to predict BMR and TDEE have been developed and validated in the able-bodied (AB) population [4,5]. However, these equations inaccurately assess energy expenditure for individuals living with SCI [5,6]. We recently reported large discrepancies between measured and estimated values of BMR that raise concern over the accuracy, reliability, and practicality of these equations in a research and clinical setting [5]. Chun et al. [7] and Nightingale and Gorgey [8], each developed an equation to estimate BMR utilizing anthropometric measures and/or DXA for those with SCI; however, these equations do not account for TEF and TEPA and are therefore unable to estimate TDEE. A commonly used equation to estimate TDEE is the product of BMR and 1.2, where 1.2 is the activity correction factor for TEF and TEPA [5,6]. Because the activity factor of 1.2 was developed for and validated in the AB population, we recently developed an SCI-specific correction factor of 1.15 to estimate TDEE [6]. The new correction factor of 1.15 offers promise for more precisely estimating their caloric intake and potentially reducing the burden of obesity and metabolic-related comorbidities in the population with SCI.

Metabolic dysfunction after SCI is marked by a greater occurrence of impaired glucose tolerance, insulin resistance, and dyslipidemia. Compared to the able-bodied population, Bauman and Spungen reported those with SCI are more likely to have insulin resistance, oral carbohydrate intolerance, elevated low-density lipoprotein cholesterol, and reduced high-density lipoprotein cholesterol. These were associated with increased frequencies of diabetes mellitus and cardiovascular disease [9,10]. The same authors also reported that 76% individuals with SCI were shown to have dyslipidemia [11], while an additional study demonstrated that 50% of those with paraplegia and 62% of those with tetraplegia had impaired glucose tolerance [12]. Moreover, in 2018, we reported that nearly 60% of veterans living with SCI were determined to have metabolic syndrome or one of its constituent components according to modified (SCI-adjusted body mass index) International Diabetes Federation criteria [3]. In addition, over 55% of these veterans were under treatment for hypertension, nearly 50% were treated for or previously diagnosed with diabetes mellitus, and about 70% were diagnosed with or under treatment for high density lipoprotein cholesterol under 40 mg/dl [3]. These results were recently supported by similar findings by Yahiro et al., in early 2019 [13]. Gorgey et al. demonstrated that total cholesterol and fasting plasma glucose were positively associated with visceral adipose tissue, supporting the hypothesis that visceral adiposity negatively influences metabolic profiles [14,15]. A previous report showed that increased trunk and leg fat and intramuscular fat contributed to insulin resistance and was predictive of plasma glucose and type II diabetes mellitus in persons with SCI [16].

Nutritional deficiencies following SCI are multifactorial, and in part stem from the obligatory sarcopenia; decreased exercise and physical activity due to the paralysis itself, a lack of adaptive exercise equipment, and transportation barriers; anabolic deficiency; and sympathetic nervous system blunting. This places the energy needs of individuals following the SCI significantly below that of the individual prior to the injury and an AB individual [4–6,17]. Therefore, a healthy diet is commonly recommended to persons with SCI because of the protective function on body composition, metabolic profiles, and overall quality of life [17–19]. However, what qualifies as a “healthy diet” for an individual living with SCI? To date, there are no evidence-based nutritional guidelines for individuals with chronic SCI, despite the presence of numerous publications outlining recommendations [17,18,20]. In an attempt to help establish evidence-based nutritional guidelines for the population with SCI, we recently published a meta-analysis and systematic review on nutritional status in chronic SCI [4]. This paper indicated greater energy intake relative to energy needs in those with chronic SCI, and an imbalance of many micronutrients compared to the United States Department of Agriculture (USDA) 2015–2020 Dietary Guidelines for Americans [4]. We showed resting metabolic rate (1492 kcal/day; CI: 1414–1569) fell below the AB average, and total energy (1876 kcal/day; CI: 1694–2059) and fiber (17 g/ day; CI: 14–20) intake were below USDA guidelines. However, despite these values coming in below the AB population, they produced a net caloric gain resulting in a positive energy balance in persons with SCI [4]. It is worthy to note, the USDA guidelines have not been validated in the SCI population. Nonetheless, individuals with SCI may not need the recommended values provided by the USDA as a result of the structural and functional changes that occur following the SCI [4]. The exact dietary requirements of individuals living with SCI warrants further research; neurogenic bowel must also be considered to ensure dietary changes do not negatively influence bowel continence. This research should focus on the caloric needs not only by measuring BMR and determining energy expenditure, but relative to body composition.

In summary, SCI is associated with many changes in body composition, metabolic syndrome and dysfunction, and overall homeostatic dysregulation that are cause for severe health concerns due to their impact on nutritional health in this special population. Looking forward, we need to establish evidence-based guidelines for individuals with SCI that are strongly rooted in clinical trials aimed at improving body composition, metabolic profiles, and nutritional health. It is essential to monitor caloric intake based on BMR and TDEE in individuals with SCI to mitigate the burden of neurogenic obesity and metabolic syndrome while concurrently correcting nutritional deficiencies that are at epidemic proportions within the SCI population [2,3].
